# Intestinal Tuberculosis

**Published:** 1901-08-10

**Authors:** 


### INTESTINAL TUBERCULOSIS.

A letter from Lord Lister has been published in which he amplifies and corrects the remarks made by him on the occasion of Professor Koch's address before the Tuberculosis Congress. The principal interest of this letter centres in what he says concerning the relation between *tabes mesenterica* and tuberculous milk. He points out that the bronchial mucus, which, together with the dust of the inhaled air, is constantly brought up to the orifice of the larynx, is perpetually swallowed, and that the inhaled dust thus as truly enters into the composition of the intestinal contents as does the food. In the case of a child fed on unboiled milk from a cow with a tuberculous udder in a room infected with tubercle it might then be fairly open to question whether the bacilli of bovine tubercle imbibed at its meals or those of human tubercle derived indirectly from the inhaled air were the more numerous in the intestine. "Hence the fact of the mesenteric glands being the only seat of tubercle in a milk-fed child is no proof that the bacilli which they contain were derived from the milk." If all this be admitted, and like everything that Lord Lister says, it carries truth and conviction on the face of it, what about the reiterated assertion that milk is responsible for the tuberculosis of infancy? The truth would seem to be that the presence of tubercle in the mesenteric glands is far more a measure of the permeability, if one may so call it, of the intestinal mucous membrane than of the bacilli present in the intestines, a permeability which is doubtless increased by anything which sets up intestinal irritation and disease. Indeed, the late Sir Richard Thorne Thorne, who was strongly convinced of the infective nature of tuberculous milk, commenting upon the remark made by Dr. Tatham that "tabes mesenterica is really more fatal during epidemic diarrhœa years," himself says, "I greatly suspect that the existence of an intestinal condition leading to diarrhœa, or the absence of any such condition, largely explains whether or not an infant, or even an adult, will be susceptible to, or experience immunity from, the infection of tuberculosis." Here we seem to get to the root of the matter. It is possible, although even that is denied by pathologists, that there may have been an increase in the amount of intestinal tuberculosis among infants of late years, or at any rate that this disease may not have diminished *pari passu* with the diminution that has taken place in other forms of tuberculosis; again, it is possible that this prevalence of intestinal tuberculosis, if it exists, may have some relation with the vast extension which has taken place of late in the practice of artificially rearing infants upon milk; and still there may be no connection whatever between the bacilli in the milk and the tubercle in the babies. Ricketty and weakly bottle-fed babies would be the best of all subjects for the development of tubercle, from whatever source the infection came—and it might just as well have come through the air as through the milk.

